# Cerebral Venous Sinus Thrombosis Linked to Dietary Supplement Use in a Bodybuilder: A Case Report

**DOI:** 10.1002/ccr3.9574

**Published:** 2024-12-31

**Authors:** Sondos K. Khalil, Zahra B. Yousif, Jawaher Baraka, Israa Al‐Hashimi, Moaz O. Moursi, Abdalla Fadul, Wanis Ibrahim

**Affiliations:** ^1^ Faculty of Medicine University of Gezira Wad Madani Sudan; ^2^ College of Medicine, QU Health Qatar University Doha Qatar; ^3^ Department of Internal Medicine Hamad General Hospital Doha Qatar; ^4^ Department of Radiology Hamad General Hospital Doha Qatar; ^5^ Weill Cornell Medical College Doha Qatar

**Keywords:** case report, cerebral venous sinus thrombosis, dietary supplements, hypercoagulability, thrombosis

## Abstract

Many dietary supplements commonly used by bodybuilders and athletes carry thrombogenic risks, potentially leading to life‐threatening conditions like arterial and venous thrombosis by either elevating testosterone levels or directly interfering with homeostasis. Increased awareness and further research are crucial for consumer safety and supplement regulation.

## Graphical abstract

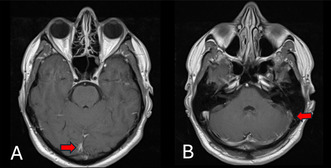

## 1 Introduction

Cerebral venous sinus thrombosis (CVST) is a life‐threatening condition, with an incidence of 1.32 cases per 100,000 person‐years [1]. CVST can manifest with diverse clinical presentations, with the most common being headaches and seizures and it can lead to serious complications, including increased intracranial pressure (ICP) and coma [2]. CVST can be caused by conditions that lead to an increased risk of blood clot formation. Examples include pregnancy, the post‐partum period, oral contraceptive use, and genetic or acquired thrombophilia. To the best of our knowledge, the association between dietary supplement use and CVST has been rarely reported in the literature [3]. In this paper, we report a rare incident of CVST associated with dietary supplements use for bodybuilding. Searching the literature, we found multiple thrombogenic ingredients in these supplements.

## 2 Case History/Examination

A 38‐year‐old gentleman was brought to our emergency department by emergency medical services (EMS) after two episodes of generalized tonic–clonic seizure, the latter was witnessed by EMS staff. His Glasgow coma scale (GCS) dropped to 8, and hence was intubated at the scene by EMS. Upon arrival at the hospital, his vitals were as follows: temperature 36.8 C, blood pressure 123/87, pulse rate 88 beats/min, oxygen saturation 98% on mechanical ventilation.

## 3 Methods (Investigations, Differential Diagnosis, and Treatment)

His initial laboratory testing showed mildly elevated creatinine with normal urea and electrolytes. Coagulation profile showed mildly increased prothrombin time and decreased activated partial prothrombin time with minimally increased antithrombin activity (Table 1). Apart from a mild increase in white blood cells, the complete blood count and liver function tests were normal. Blood toxicology screening for ethanol and acetaminophen levels was negative.

**TABLE 1:** Initial basic blood investigations.

Variable	Result	Reference range
WBC	17.8 × 10^3^/uL	4–10
Hgb	14.3 g/dL	13–17 mg/dL
MCV	87.4	83–101
Platelets	220 × 10^3^/uL	150–410
PT	12.7 s	9.7–11.8 s
APTT	21.7 s	24.6–31.2 s
INR	1.2	Critical high > 4.9
Creatine	134 μmol/L	62–106 μmol/L
Urea	4.5 mmol/L	2.5–7.8 mmol/L
Sodium	138 mmol/L	133–146 mmol/L
Potassium	4.7 mmol/L	3.5–5.3 mmol/L
Adjusted calcium	2.34 mmol/L	2.20–2.60 mmol/L
Magnesium	0.96 mmol/L	0.7–1.00 mmol/L
ALT	19 U/L	0–41
AST	21 U/L	0–40
ALP	119 U/L	40–129
Albumin	37 g/L	35–50
Total Protein	73 g/L	60–80
Total bilirubin	4 Umol/L	0–21
CRP	5.3 mg/L	0–5
Ethanol level	< 2.2 mmol/L	
Acetaminophen level	Negative	

Abbreviations: ALT, alanine transaminase; APTT, activated partial thromboplastin time; AST, aspartate transaminase; CRP, C‐reactive protein; GGT, gamma‐glutamyl transferase; Hgb, hemoglobin; INR, international normalized ratio; MCV, mean corpuscular volume; PT, prothrombin time; WBC, white blood cell count.

Electrocardiogram, chest X‐ray, and an urgent computed tomography (CT) scan of the head were unremarkable. He was admitted to the Medical Intensive Care Unit (MICU) and the next morning his GCS improved to 15/15 and he was extubated.

History taken after the patient awakened revealed a healthy young gentleman with no comorbidities. He reported that this was the first time he had a seizure. He denied a history of headaches, loss of consciousness, speech disorder, swallowing problems, motor weakness, lack of coordination, or involuntary movements. He was not on any medication. He has never smoked cigarettes, consumed alcohol, or used illicit drugs. Family history was unremarkable. Systematic review was also unremarkable. Examination revealed normal neurological, abdominal, respiratory, and cardiovascular systems.

**FIGURE 1:**
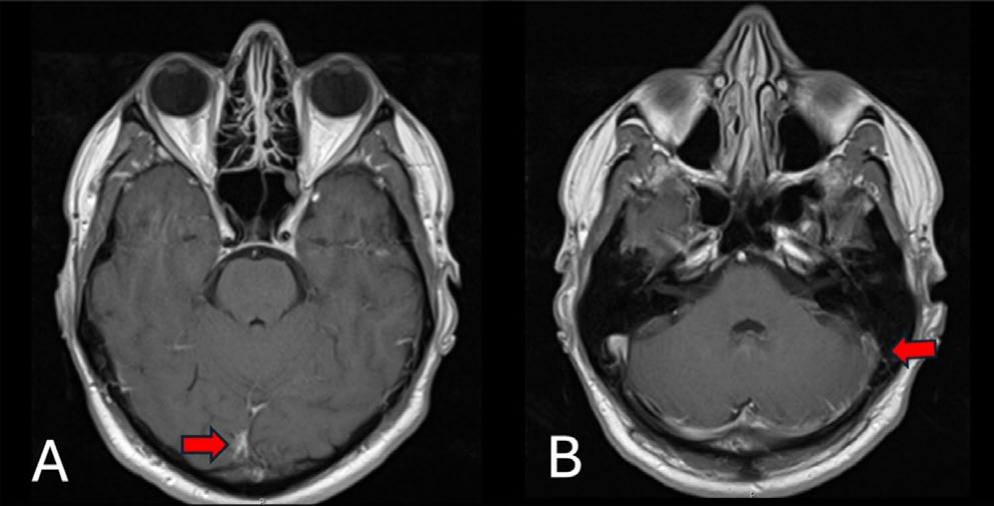
(A) T1‐post contrast axial MRI showed very thin central filling defect (the red arrow) along the superior sagittal sinus and (B) T1‐post contrast axial MRI showed filling defect (the red arrow) along the left transverse and sigmoid sinuses as well as the internal jugular vein denoting acute sinus thrombosis.

**FIGURE 2:**
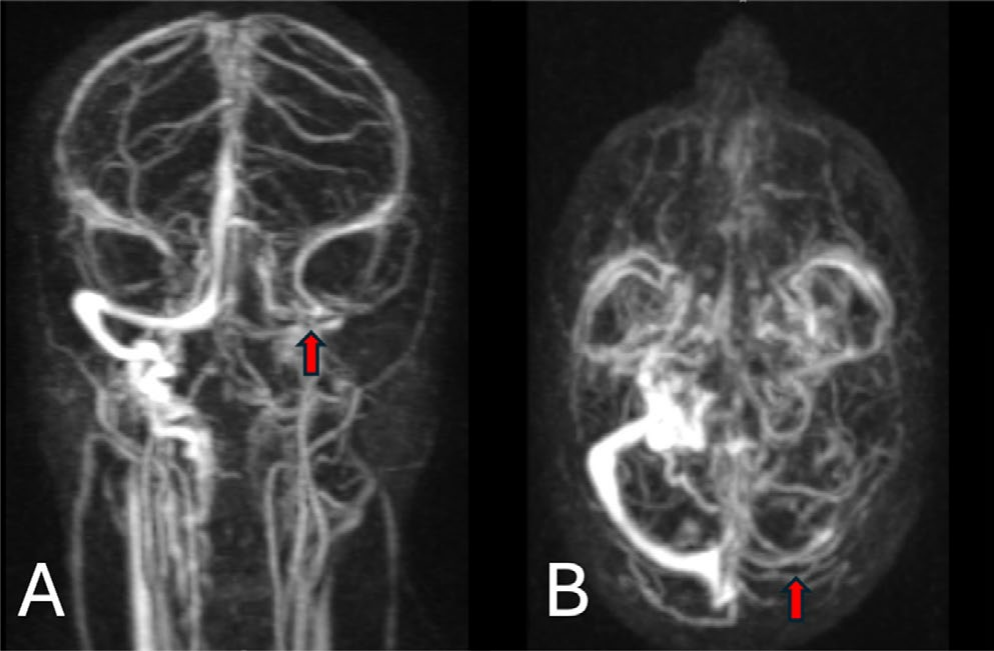
(A) TWIST Coronal MRV‐MIP and (B) TWIST axial MRV‐ MIP showed complete absence of the left transverse and sigmoid sinuses and left internal jugular vein (the red arrows) denoting for acute thrombosis.

**FIGURE 3:**
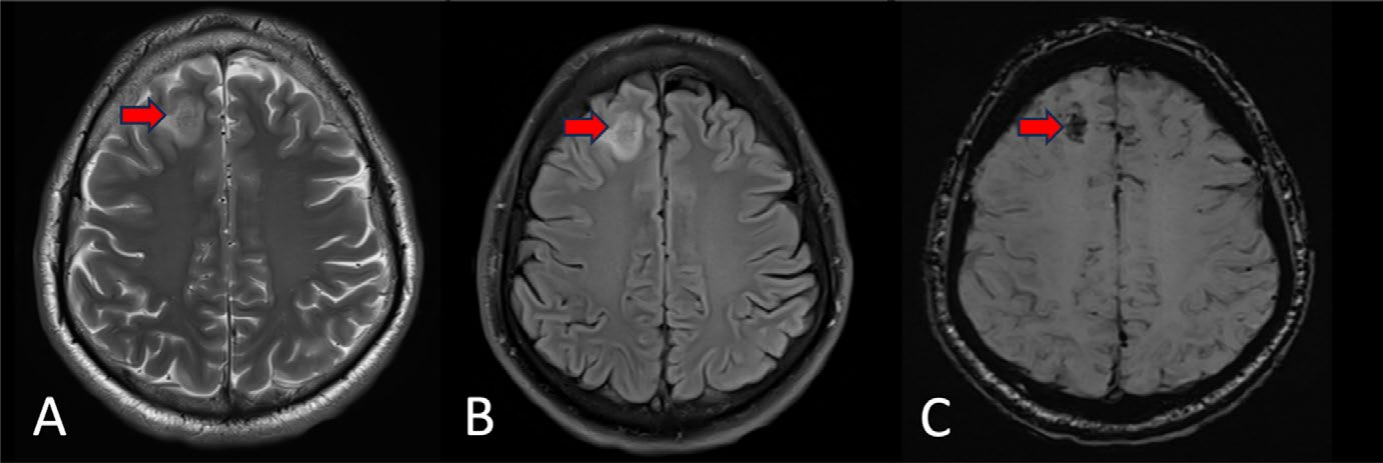
(A) T2‐TSE‐AXIAL MRI and (B) T2‐TSE‐FLAIR (dark fluid) MRI showed right high frontal cortical/juxta‐cortical lesion with mixed signal intensity and surrounding vasogenic edema, (C) SWI‐axial MRI showed extensive abnormal blooming (the red arrows) denoting for hemorrhagic component.

**FIGURE 4:**
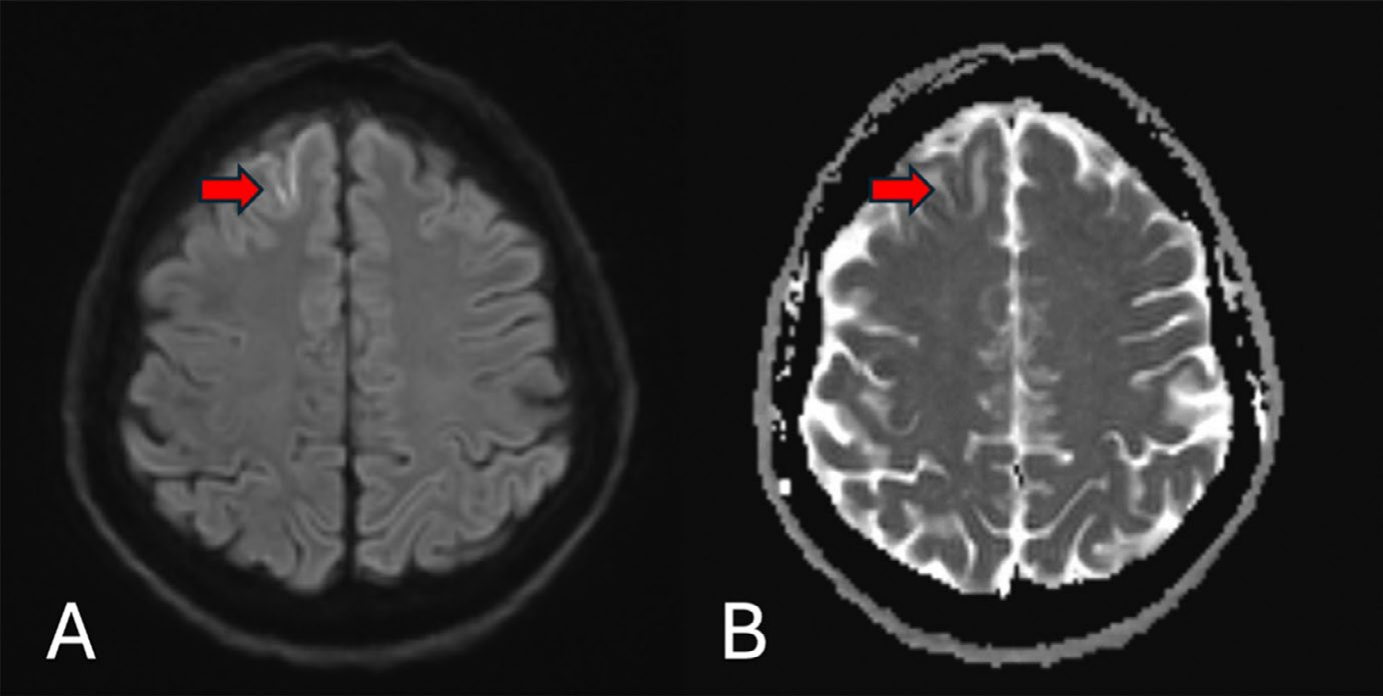
(A) DWI axial MRI and (B) ADC axial MRI showed gyriform pattern diffusion restriction (the red arrows).

Magnetic resonance imaging (MRI) and magnetic resonance venography (MRV) of the head with contrast showed features of superior sagittal as well as left sigmoid and transverse sinuses thrombosis (Figures 1 and 2). A small right high frontal venous hemorrhagic infarct was also noted (Figures 3 and 4).

Further testing done to identify the underlying pathology showed an insignificant increase in antithrombin activity, normal fibrinogen, protein C, and protein S activities, with normal tumor markers for testicular cancer. The rest of the thrombophilia work‐up was also negative (Table 2).

**TABLE 2:** Thrombophilia work‐up.

Variable	Result	Reference range
Fibrinogen	3.91 g/L	2.0–4.20 g/L
Protein C activity	135.5%	70%–140%
Protein S activity	117.0%	72%–126%
Factor V Leiden mutation	Negative	
ATAC	119.6%	79.4%–112%
ANCA	Negative	
Lupus anticoagulant	Not detected	
Anticardiolipin IgG	2.00 GPL	15–20 GPL
Anticardiolipin IgM	1.20 MPL	12.5–20 MPL
Anti‐beta‐2 glycoprotein IgG	0.90 U/mL	< 20 U/mL
Anti‐beta‐2 glycoprotein IgM	< 2.90 U/mL	< 20 U/mL
AFP	1 IU/mL	0–6 IU/mL
Beta‐hCG	< 1 mIU/mL	0–2 mIU/mL
Jak2 V617F missense mutation	Negative	

Abbreviations: AFP, Alpha‐fetoprotein; ANCA, Antineutrophilic cytoplasmic antibody; ATAC, antithrombin activity; Beta‐hCG, Beta‐Human Chorionic Gonadotropins;IgG, Immunoglobulin G; IgM, immunoglobulin M.

He was started on therapeutic enoxaparin (100 mg twice daily) and valproic acid (500 mg twice daily), stayed in the MICU for 4 days under observation, then was shifted to the medical ward; however, no clear cause could be identified. Upon conducting a more comprehensive history, the patient disclosed the use of three dietary supplements for bodybuilding at the recommended dose by the manufacturer, started 10 days prior to admission. The supplements' labels/ingredients as stated by the manufacturer are listed in Table 3.

**TABLE 3:** Supplements' labels/ingredients as stated by the manufacturer.

Ingredients	Amount per serving	DV%
Supplement 1 (enhanced athlete blue ox)		
Vitamin D3 (as cholecalciferol) (1600 IU)	40 mcg	200
Vitamin E (as D‐alpha tacopherol succinate) (200 IU)	134 mg	890
Magnesium (as magnesium aspartate)	400 mg	100
Zinc (as zinc aspartate)	20 mg	180
Bindii Extract (*Tribulus terrestris*) (fruit) min. 45% saponins (225 mg)	500 mg	^a^
Nettle extract (*Urtica dioica*) (leaf)	500 mg	^a^
Ashwagandha extract (*Withania somnifera*) (root)	300 mg	^a^
Shilajit extract (*Asphaltum* spp.) (fossilized plant) min. 40% fulvic avid (80 mg)	200 mg	^a^
Borogonic glycine	5 mg	^a^
Supplement 2 (enhanced athlete slin)		
Chromium (as chromium chelate)	200 mcg	167
Berberine HCI (*Berberis aristate*) (root, stem, and bark)	500 mg	^a^
Bitter melon extract (*Momordica charantia*) (fruit)	300 mg	^a^
Cinnamon extract (*Cortex cinnamon*) (bark)	200 mg	^a^
Alpha lipoic acid	150 mg	^a^
Fenugreek extract (standardized to 50% saponins) (*Trigonella foenum‐graecum* L.) (seeds)	150 mg	^a^
Kaempferol 10% (*Sophora japonica*) (fruit)	150 mg	^a^
Bayberry bark extract (70% Myricetin) (bark)	90 mg	^a^
Banaba extract (*Lagerstroemia speciosa*) (leaf)	90 mg	^a^
African mango extract (*Irvingia gabonensis*) (seed)	90 mg	^a^
Fucoxanthin 10% (*Laminaria japonica*) (seaweed)	8 mg	^a^
Supplement 3 (enhanced athlete arachidonic acid)		
Arachidonic acid	1400 mg	^a^

^a^ Daily Value not established.

## 4 Conclusion and Results (Outcome and Follow‐Up)

A diagnosis of CVST secondary to bodybuilding dietary supplements use was reached. The patient was discharged on apixaban (5 mg twice daily for 3 months) and valproic acid (500 mg twice daily), and was advised against the use of the supplements. On follow‐up, the patient reported feeling well and had been symptom‐free for a year. Repeated MRI and MRV showed recanalization of the sinuses.

## 5 Discussion

Dietary supplements have been used in sports for several purposes, including treating sport‐associated injuries, recovering from sports fatigue [4, 5], and increasing muscular strength and muscle mass [6, 7]. Dietary supplementation has been associated with thrombosis, as reported in several studies [8, 9, 10]. Although CVST linked to dietary supplementation is rarely described in the literature, it has been primarily attributed to the use of anabolic‐androgenic steroids [11]. After a careful review of the literature, we identified only one case of CVST in a patient taking multiple dietary supplements as part of a naturopathic anti‐aging regimen [3]. Our patient consumed three bodybuilding supplements, namely: enhanced athlete blue ox, enhanced athlete slin, and enhanced athlete arachidonic acid. Following an in‐depth literature review of these supplements' ingredients (Table 3), our analysis has revealed that these ingredients increase the risk of thrombosis through two mechanisms. First, by increasing testosterone levels which in turn causes a prothrombotic state [12], and second, by directly interfering with factors that regulate homeostasis, leading to CVST [12].

All components of supplement 1 (enhanced athlete blue ox) and supplement 3 (enhanced athlete arachidonic acid) have demonstrated the capacity to increase testosterone levels in human and animal subjects [13, 14, 15, 16, 17, 18, 19]. Vitamin D is an exception as conflicting research studies regarding its impact on testosterone level exist, with some studies suggesting the ability to elevate testosterone [20], while others suggest the opposite [21]. All components of supplement 2 (Enhanced Athlete Slin) with the exception to Kaempferol, and Bayberry Bark Extract (70% Myricetin) have also been proven to increase testosterone in humans and animals [22, 23, 24, 25, 26, 27, 28, 29].

These components can increase testosterone levels by different mechanisms, for instance, Fenugreek seed extracts were suggested to increase free testosterone either by decreasing its metabolism or acting as aromatase and 5α‐reductase inhibitors [17, 30]. Ashwagandha acts by up‐regulating gonadotropin‐releasing hormone (GnRH) and in turn testosterone production by Leydig cells [17]. Nettle (*Urtica dioica*) leaves have shown to increase testosterone bioavailability by inhibiting its binding to sex hormone‐binding globulin [31]. The exact mechanism by which the rest of the components can increase testosterone levels remains unclear and not yet fully understood. Dietary therapy can directly disrupt hemostasis by altering factors responsible for homeostasis regulation. For instance, arachidonic acid was noted to enhance platelet aggregation in cirrhotic patients with low platelet counts [32], although the exact mechanism remains incompletely understood.

Despite having several prognostic factors typically associated with poor outcomes, such as impaired consciousness at presentation (GCS 3–8), seizure at onset, multiple sinus thrombosis, male sex, and age ≥ 30 years [33, 34, 35], interestingly, our patient achieved a complete recovery, with a modified Rankin scale of 0.

In conclusion, dietary supplements used by athletes and bodybuilders can create a thrombogenic state by increasing testosterone levels or directly interfering with homeostatic factors and can potentially lead to arterial and venous thrombosis. Further studies and research are needed to determine adequate concentrations measured against the duration of therapy to ensure the safety of these supplements while maintaining their efficacy.

## Author Contributions

**Sondos K. Khalil:** conceptualization, data curation, writing – original draft. **Zahra B. Yousif:** conceptualization, data curation, writing – original draft. **Jawaher Baraka:** conceptualization, data curation, writing – original draft. **Israa Al‐Hashimi:** conceptualization, data curation, writing – original draft. **Moaz O. Moursi:** writing – review and editing. **Abdalla Fadul:** conceptualization, data curation, writing – original draft. **Wanis Ibrahim:** supervision, writing – review and editing.

## Ethics Statement

All methods were performed in accordance with the relevant guidelines and regulations. Written informed consent was obtained from the patient.

## Consent

Written informed consent was obtained from the patient for publication of this case report and any accompanying images. A copy of the written consent is available for review by the Editor‐in‐Chief of this journal on request.

## Conflicts of Interest

The authors declare no conflicts of interest.

## Data Availability

The authors have nothing to report.
